# Spiral Modes and the Observation of Quantized Conductance in the Surface Bands of Bismuth Nanowires

**DOI:** 10.1038/s41598-017-15476-5

**Published:** 2017-11-14

**Authors:** Tito E. Huber, Scott Johnson, Leonid Konopko, Albina Nikolaeva, Anna Kobylianskaya, Michael J. Graf

**Affiliations:** 1 Howard University, Washington, DC 20059 USA; 2 Academy of Sciences, Chisinau, MD-2028 Moldova; 3 International Laboratory of High Magnetic Fields and Low Temperatures, 53-421 Wroclaw, Poland; 4 Department of Physics, Boston College, Chestnut Hill, MA 02467 USA

## Abstract

When electrons are confined in two-dimensional materials, quantum-mechanical transport phenomena and high mobility can be observed. Few demonstrations of these behaviours in surface spin-orbit bands exist. Here, we report the observation of quantized conductance in the surface bands of 50-nm Bi nanowires. With increasing magnetic fields oriented along the wire axis, the wires exhibit a stepwise increase in conductance and oscillatory thermopower, possibly due to an increased number of high-mobility spiral surface modes based on spin-split bands. Surface high mobility is unexpected since bismuth is not a topological insulator and the surface is not suspended but in contact with the bulk. The oscillations enable us to probe the surface structure. We observe that mobility increases dramatically with magnetic fields because, owing to Lorentz forces, spiral modes orbit decreases in diameter pulling the charge carriers away from the surface. Our mobility estimates at high magnetic fields are comparable, within order of magnitude, to the mobility values reported for suspended graphene. Our findings represent a key step in understanding surface spin-orbit band electronic transport.

## Introduction

**Figure 1:**
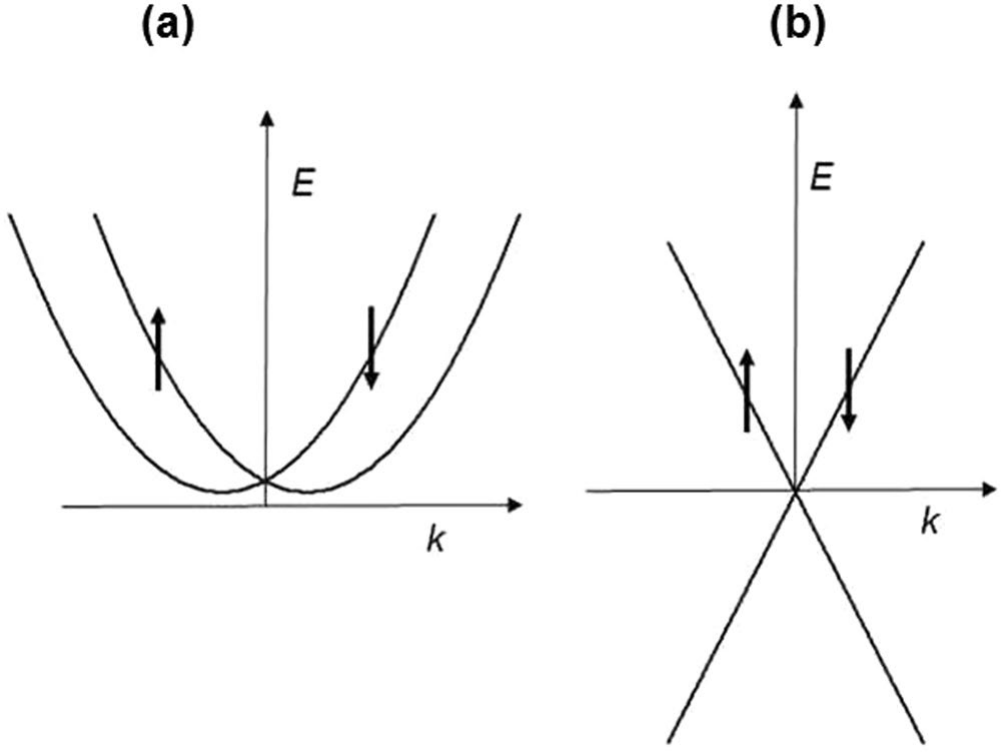
Surface bands energy dispersion owing to spin-orbit coupling. (**a**) Spin-split bismuth. (**b**) Topological insulator surface states exhibiting Dirac energy dispersion and spin locking.

Surface bands that appear as a result of surface spin-orbit coupling (SOC) represent a new direction in the field of two-dimensional (2D) electron gases. Researchers have observed the SOC surface bands via angle-resolved photoemission spectroscopy (ARPES) in many materials, including bismuth^1,2^ and topological insulators (TIs)^2,3^ such as Bi_2_Se_3_ and Bi_2_Te_3_. From ARPES, many details of SOC bands have been revealed, such as the Fermi energy, Fermi wavenumber, energy dispersion and surface charge density. The behaviour of these bands is different from the behaviours described in the well-studied cases of semiconductor interfaces and graphene^4^ because the electron motion is uniquely spin polarized. The SOC bands in Bi, which are spin split, and TI bands, which exhibit spin locking, are illustrated in Fig. 1. Two important features of 2D electron gases are very high electronic mobility and quantum electronic transport, as in the case of graphene. The Landauer-Büttiker formula^5^ *G* = *G*_0_ *MT* relates the conductance *G* of a quantum conductor to the electronic bands and their scattering properties, where *e* is the electron charge; *h* is Planck’s constant; *M* is the number of sub-bands or transverse modes in the wire at the Fermi level; *T* is the transmission, which is between 0 and 1. *G*_0_ = 2*e* ^2^/*h* is the quantum conductance, which is equal to 7.75 × 10^−5^ Ω^−1^. Step-like changes in the conductance, which signify abrupt changes in *M*, follow from the Fermi level crossing of the sub-band energy levels induced by an applied field. Experiments have been performed with short graphene strips (length *l* ~ 10 nm), shorter than the transport mean free path (*l*_*e*_) in the wire, where the effect is cleanly manifested by the steps of the conductance^6^ with the magnitude *G*_0_. If, however, *l*≫ *l*_*e*_, because to satisfy Ohm’s law, *T* = *l*_*e*_ /(*l* + *l*_*e*_), then the steps that are observed are only 2*e* ^2^ (*l*_*e*_/(*l* + *l*_*e*_)) /*h*. Such stepwise conductance is observed in long strips of graphene^7,8^. Here, we show that the SOC bands in 50-nm Bi nanowires exhibit stepwise conductance with a long mean free path (19 μm), indicating that these bands have very high mobility *μ*. The mobility *μ* = 2π*el*_*e*_/*hk*_F_, where *k*_F_ is the Fermi wavenumber, is found to be exceptionally high, rivalling that of graphene.

The surface-to-volume ratio of our nanowires is high, and, therefore, surface effects are strongly expressed. Also, quantum confinement reduces the bulk carrier density^9^ thereby further increasing the contribution of the surface bands to nanowire electronic transport. Conductance studies of small-diameter Bi nanowires^9,10^ and nanoribbons^11^ clearly reveal surface conduction. Confinement of the electron gas along the circumference gives discretely quantized circumferential momentum and generates a series of one-dimensional (1D) surface modes. Under an applied magnetic field *B* parallel to the wire axis, the surface electronic wave function picks up the Aharonov–Bohm (AB)^12–14^ phase of 2π*Φ*/*Φ*_*0*_, where *Φ* is equal to the total magnetic flux through the cross-sectional area and *Φ*_0_ = *h*/*e* (the magnetic flux quantum). In experiments using small-diameter nanowires (the result of recent advances in fabrication techniques, see Supplementary Information), we observed marked *h/e* and *h/2e* modulation of the conductance *G*(*B*). *G* exhibited the steps and plateaus that we interpret to be quantum conductance. It was clear that the pattern of oscillations in the magnetoresistance is related to high-mobility modes. The model that we propose for coexisting *h*/*e* and *h*/2*e* periods involves spiral states, which are modes that have been observed in the high-mobility quasiballistic conduction bands of semiconductor nanowires^15–17^. Spiral states have not previously been investigated for Bi SOC surface states. In spiral states spin-split energy bands, the *h*/2*e* periodicity can arise because there are pairs of levels that cross the Fermi level during each cycle of the AB phase. A spiral state’s rotational energy has the following dispersion:

1 $${{E}}_{{s}}^{{L}}{\rm{=}}\frac{{{h}}^{{2}}}{{2}{\pi }^{{2}}{{m}}_{{\Sigma }}{{d}}^{{2}}}{({L}{-}\frac{{\Phi }}{{{\Phi }}_{{0}}})}^{{2}}{\rm{+}}{g}{\mu }_{{B}}{B}{s}$$

The rotational energy depends on the orbital quantum number *L*, which is the angular momentum in the direction along the wire axis and the spin direction *s*, where *s* is +1 and −1 for spin-up and spin-down, respectively and the wire diameter *d*. The effective mass *m*_Σ_, in units of the electron mass, is the in-plane effective mass^15^. The second term is the Zeeman energy, where *g* is the electron spin factor and *μ*_B_ is the Bohr magneton, that is, 5.8 × 10^−5^ eV/T. The dispersion relation in equation (1) is reminiscent of the one for helical states that are observed in TIs^18–20^ but there are significant differences that we discuss below. The total energy includes the kinetic energy of motion along the wire axis. In this work we present evidence for the existence of subsurface spiral modes of SOC surface states in Bi nanowires. We then show that these modes exhibit quantized conductance and discuss the surface model that explains the observations.

## Results

**Figure 2:**
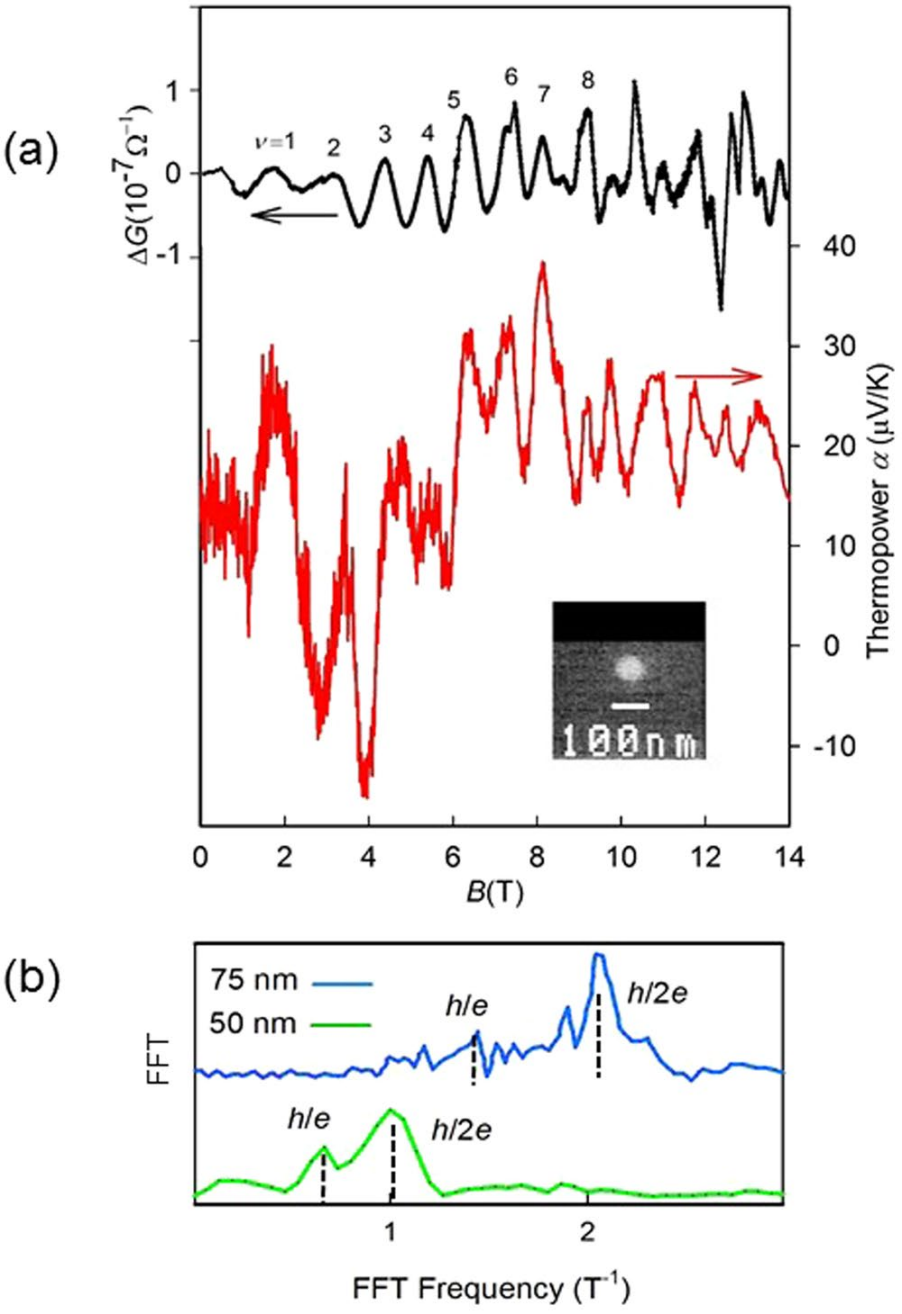
Aharonov–Bohm oscillations in a 50-nm Bi nanowire. (**a**) Black and red represent Δ*G* and thermopower *α*, respectively, as a function of *B* along the wirelength of sample Q1 at 1.5 K. Δ*G* is the conductance minus a smooth background. The minima order *ν* is indicated. Inset, SEM cross-sectional image of the (50 ± 5)-nm wire (clear) in its glass envelope (grey background). (**b)** FFT of *G* of 50- and 75-nm wires, as indicated, in the entire field range (0 T–14 T). Vertical dashed lines indicate the *h*/*e* and *h*/2*e* peaks.

The diameters of the samples of single-crystal Bi nanowires used in our experiment ranged between 45 nm and 55 nm. These were fabricated in several stages (see Methods section and also Supplementary Figure 1). The wires are long (fractions of a millimetre), and the contact resistance (of order kΩ) is much less than the zero-field resistance. Supplementary Figure 2 shows scanning electron microscope images of the cross-section. As shown in Supplementary Figures 3–7 we characterized the temperature dependence of the resistance and the dependence of the magnetoresistance with the orientation of the nanowires with the magnetic field. We observed a thermally activated conductivity that is typical of semiconductors at *T* > 100 K, which is different from that of bulk semimetallic Bi due to confinement effects^9^. At low temperatures, *T* < 10 K, the conductivity becomes saturated, indicating electronic transport by surface carriers. The samples exhibited sharp AB oscillation (periodic in *B*), and one particular sample (Q_1_), with a diameter of 50 nm, was selected for thermopower experiments. In Fig. 2, we present magnetoconductance Δ*G*, with the smooth background subtracted, and also the thermopower *α*, with applied field *B* at 1.5 K. The sample length *l* of Q_1_ was 500 ± 100 µm. We show similar transport results for samples Q_2_ and Q_3_ in the Supplementary Information. For sample Q_1_, *G* and *α* measurements were performed together, in the same experimental device, using a 15 T superconducting magnet. The value of *G* at low temperature was 2.8 × 10^–6^ Ω^−1^. The thermopower output was positive, indicating that the charge carriers are holes. A scanning electron microscope (SEM) image of the wire cross-section is shown in the inset of Fig. 2. Errors in *d* were 10% because the nanowire was immersed in the glass fibre, and the glass became charged, thereby reducing the resolution of the SEM.

As shown in Fig. 2, we observed a deep periodic modulation of the conductance *G* and thermopower with applied *B*. Two *G*(*B*) oscillation periods of 0.98 ± 0.05 T and 1.7 ± 0.1 T were detected, with the faster oscillation being the more visible for *B* > ~3 T. The parameter *ν* is the index of the minima of oscillations. A fast Fourier transform (FFT) of the data, presented in Fig. 2b, reveals broad peaks at 1.0 T and 1.7 T, corresponding to the slow and fast oscillations, respectively. For comparison, Fig. 2b also shows the FFT in the case of a 75-nm nanowire^9^. The observed periods are consistent with *h/e* and *h/2e* periods in 50-nm nanowires within the experimental errors in the diameter^21^ and the AB periods. The modulation (Δ*G*/*G* ~ 0.1) was deep. Here the oscillatory component Δ*G* is obtained from *G* by subtracting a smooth background. Using the width of the spectrum at *h*/*e* as an estimate for the uncertainty of oscillation frequencies, and assuming that the spread in the frequencies results from the carriers’ occupation of a region of finite width *w* near the surface (i.e., a spread in orbital radii), we estimate *w* ~ 5 nm; because the spectral width can also be augmented by a variation in the wire diameter along its length, this estimate is an upper bound for *w*. The uncertainty of oscillation frequencies can also be partially attributed to the Zeeman energy in equation (1)^15^. The (*B* = 0) low temperature conductance that we observed corresponds to a sheet resistance of 186 Ω/□. This value is comparable to the room temperature value of 660 Ω/□ previously observed in ultrathin (2.5-nm) Bi films that feature high-mobility surface charges^22,23^.

We tested the surface via a study of the Shubnikov-de Haas (SdH) oscillations (periodic in 1/*B*) of the transverse magnetoresistance (TMR), where the field is perpendicular to the long axis of the wire. We considered the presence *h*/*e* and *h*/2*e* oscillations to be strong evidence of the 2D character of the surface, and, therefore, we assigned the SdH oscillations to 2D Landau states of the surface carriers. Analysis of the temperature and magnetic field dependence of the SdH oscillations^24,25^, described in detail in the Supplementary Information, produced *m*_Σ_ = 0.25 ± 0.03 in units of the electron mass *m*_0_, in good agreement with ARPES measurements^1^. See Supplementary Figures 8 and 9. The charge density per unit area Σ was estimated from the SdH period (*P* = 0.060 *T* ^−1^) using Σ = *f*/(*PΦ*_*0*_), where *f* is the 2D Landau level degeneracy^4^, which is two on account of the two-fold spin degeneracy. We found Σ = 8.06 × 10^11^/cm^2^, which was an order of magnitude smaller that the ARPES measurement^26^ of 8 × 10^12^/cm^2^ for Bi crystals. The 2D Fermi energy *E*_F_ = ${\rm{\pi }}{\hslash }^{2}{\Sigma }/{m}_{{\rm{\Sigma }}}$ and *k*_F_ were found to be 7.6 meV and 2.2 × 10^8^/m, respectively. Taking Σ to be 8 × 10^12^/cm^2^ (ARPES value), we estimated *E*_F_ = 76 meV and *k*_F_ = 7.1 × 10^8^/m. The analysis of SdH oscillations was not straightforward because of the cylindrical geometry of the wires. Treating this as a flat surface perpendicular to the field was obviously not valid for the geometry of a nanowire because most of the surface was not perpendicular to the magnetic field, even if the field was perpendicular to the wire axis^27^. Therefore, we considered the value of Σ from ARPES to be more appropriate for our nanowires than the value estimated from SdH oscillations.

**Figure 3:**
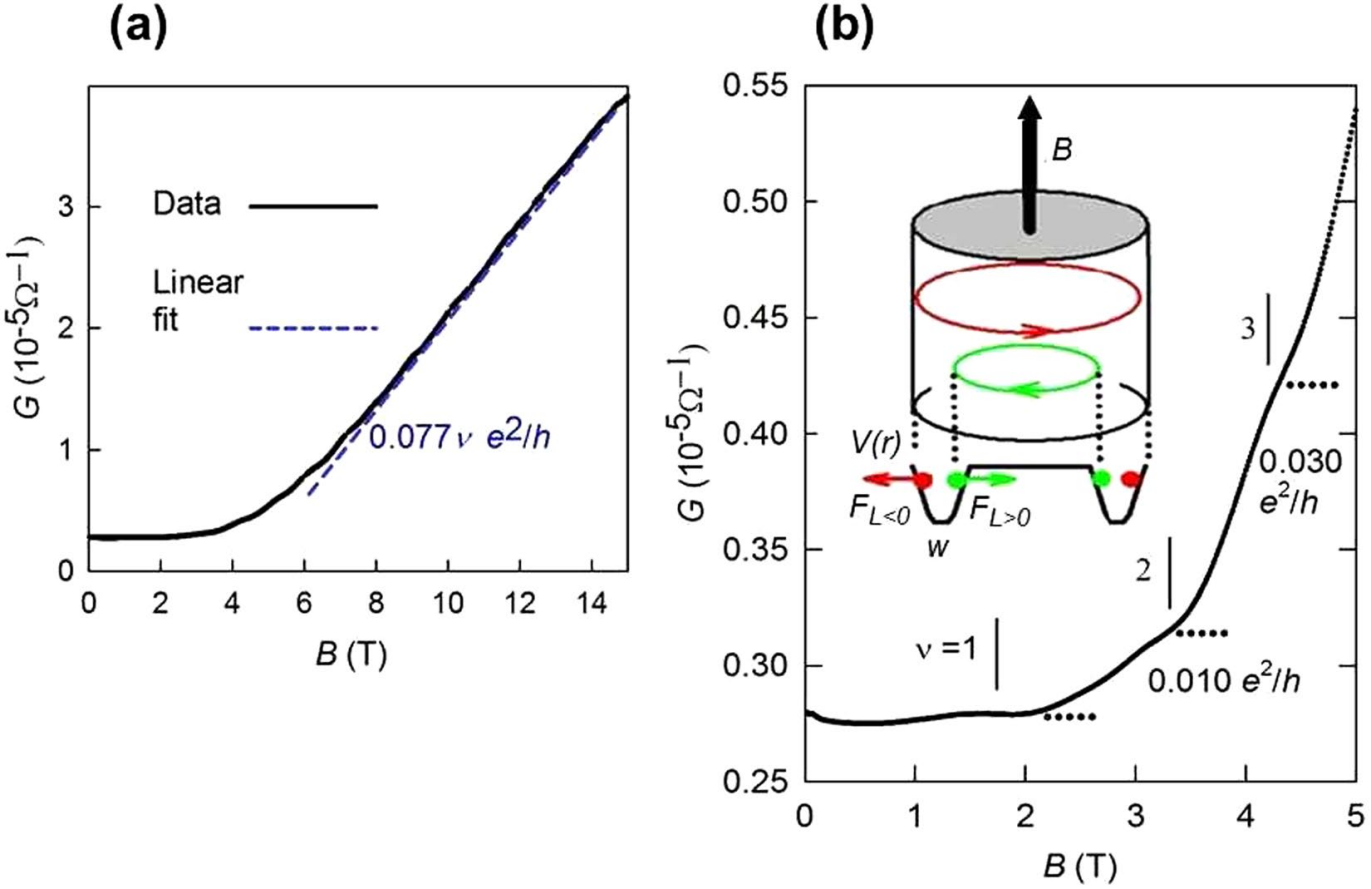
Conductance of the 50-nm Bi nanowire. (**a**) *G* of sample Q1 as a function of *B* measured at 1.5 K. The dashed line represents the linear fit *G*_*W*_ *ν*, where *G*_*W*_ = 3.0 × 10^−6^ Ω^−1^ and *ν* = *B* (0.98 T)^−1^. (**b**) The scale for *G* is expanded so as to make the conductance steps at *ν* = 1, 2 and 3 evident. The plateaus are indicated with horizontal lines, and the values of the steps of *G* are indicated. Inset. Illustration of the nanowire encircled by surface holes in high-mobility (green) and low-mobility (red) orbits based on our estimate for the effect of the Lorentz force. The toroidal subsurface confinement potential *V*(r) of surface range *w* and the Lorentz forces (*F*_*L*>0_ and *F*_*L*<0_) are also shown.

Figure 3 shows the conductance *G* of the 50-nm sample Q_1_ at 1.5 K as a function of the magnetic field applied parallel to the long axis of the wire. For high magnetic fields, *G* increased by more than one order of magnitude, from *B* = 0 T to 15 T. We presume the high value of *G* observed at 14 T, *G* = 3.88 × 10^−5^ Ω^−1^, to be limited by the contact resistance of 2.5 kΩ. Plateaus are observed in *G* in Fig. 3b. The plateaus in *G* are shown for *ν* = 1 to 3, where *ν* can be interpreted as the number of surface conduction channels. We observed that *G*_W_ increased with every increment of *ν*. For larger values of *B*, *B* > ~6 T, *G* increased almost linearly with *B*. If we consider the period of the oscillation to be *h/2e* = 0.98 T, then the linear increase of *G* implies a constant step value of *G*_W_ = 3.0 × 10^−6^ Ω^−1^ for *ν* > ∼ 5 (*B* > ∼7 T). Plateaus and the linear dependence of the conductance fit a conduction model based on the cyclic opening of 1D channels. According to the Landauer formula^5^, *G* = *G*_W_ *M*, where *G*_W_ = (2*e* ^2^/*h*)(*l*_*e*_/*l*). Associating *M* with *ν*, our estimate for maximum *l*_e_ was 19 μm, which was almost 400 times the wire diameter. Correspondingly, the mobility *μ* = 2π*el*_*e*_/*hk*_F_ increased with the magnetic field, and the highest observed value was estimated to be 410,000 cm^2^V^−1^s^−1^, using the *k*_F_ value obtained via ARPES. We found that *μ* can also be estimated directly from the nanowire conductance using *μ* = *G*/*Σ*. This estimate depends on *B* because *G* increases with the magnetic field. For *B* = 0 T, we found *µ* = 14,000 cm^2^V^−1^s^−1^, and for *B* ~ 10 T, we found *µ* = 130,000 cm^2^V^−1^s^−1^; this value represents a lower bound because the measured high field conductance is limited by the contact resistance. These findings confirm the high value of the estimate based on Landauer’s expression and also indicate that the surface band mobility increases with increasing *B* for *B* < 7 T, stabilizing at the high value for *B* > 7 T up to the maximum value of *B* in our measurement. Our mobility estimates are comparable, within an order of magnitude, to the *μ* values reported for suspended graphene^28^ (in excess of 600,000 cm^2^V^−1^s^−1^).

**Figure 4:**
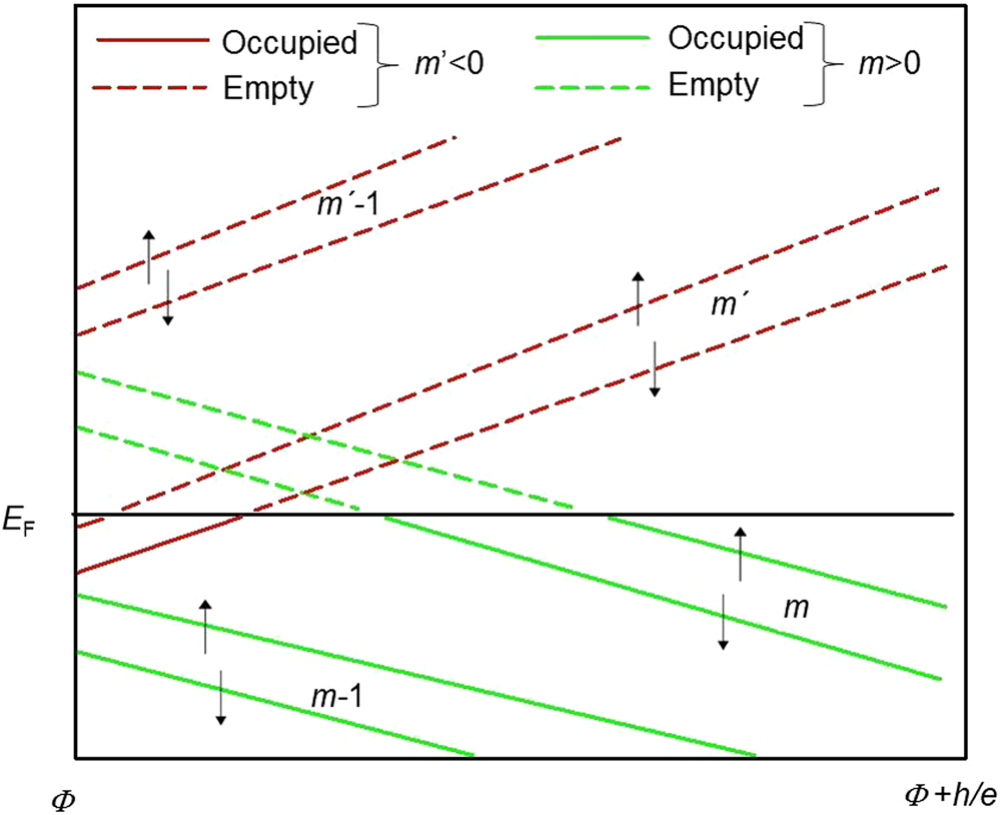
Spiral sub-bands crossing the Fermi level. Spiral sub-bands are calculated using equation (1) in the *L* > 0 and *L* < 0 cases, after setting *g* = 2. *m* and *m*’ are the special angular momenta *L* that lead to level crossings in the range between a given *Φ* and *Φ* + *h*/*e*. The *E*_F_ is indicated by the black solid line.

We interpret the observations, including the presence of *h*/*e* and *h*/2*e* periods, by considering spiral modes, specifically equation (1). At low temperatures, only the sub-bands with ${E}_{s}^{L} < {{E}}_{{\rm{F}}}$ are occupied. For *B* = 0 T, assuming that *E*_F_ = 7.6 meV (SdH value), the sub-bands with $|L|$ < 6 are occupied. If we assume that *E*_F_ = 76 meV (ARPES value), then the maximum value of $|L|$ is 18. In either case, the cyclic AB phase imposes the same constraints. Under the applied field *B*, there are pairs of sub-band levels crossing the Fermi level (${E}_{s}^{L}$ = *E*_F_) per AB cycle, that is, for the flux change ${\Delta }{\Phi }$ = *h*/*e*. This result is illustrated in Fig. 4. At a level crossing the nanowire (translational) kinetic energy is zero and a van Hove singularity appears. Sub-bands exhibit a complex behaviour that tends to keep the total carrier density constant. With increasing *B*, ${E}_{s}^{L}$ increases for the sub-bands with negative *L* and decreases for the sub-bands with positive *L* (Equation 1). With increasing *B*, four Fermi level crossings are found per AB cycle, that is, for ${\Delta }{\Phi }$ = *h*/*e*. For each cycle, a pair of states with negative *L* of opposing spin orientations are transformed from occupied to empty, and a pair of states with positive *L* are transformed from empty to occupied. The opening/closing of the 1D channel gives rise to positive/negative steps in *G*. Thus, there are two pairs of peaks in the Δ*G* and two steps in the conductance The presence of pairs of level crossings per cycle can explain the presence of both *h*/*e* and *h*/2*e* periods.

We also observed (see Fig. 3) that *G* increased with increasing *B* and *ν*. Because the increase in *B* caused the number of modes with negative *L* to increase, we found the modes with positive *L* to be high-mobility modes and that the mobility of the modes with negative *L* is low. This effect can be interpreted by considering that the surface charges are holes; assuming that the holes are confined to the surface potential well, the Lorentz force tends to move the orbit of the modes with positive *L* closer to the surface of the Bi nanowire while pushing the modes with negative *L* away from the surface, as illustrated in the inset of Fig. 3b. The modes with negative *L* experience more bulk-like conditions than the modes with positive *L*. All other factors being equal, the proximity to the surface can decrease mobility via surface scattering. Note that the surface of Bi is not a simple sharp 2D interface. It has been shown, via ARPES measurements coupled with theoretical studies^29^, that some surface states penetrate deeply, a few nanometres, into the bulk. This is close to our estimate of *w*, 5 nm, from the spread in the *h*/*e* frequencies. Moreover, scanning tunnelling microscopy experiments showing that the Bi bilayers carry one-dimensional topological edge states^30^, suggest that the surfaces of Bi are not sharply terminated, providing support for the case of surface penetrating states. The potential well model has been proposed to explain confinement of charges in rings that support *h*/*e* and *h*/2*e* oscillation^31^. The observed effect, that entails negative magnetoresistance is similar to the Chambers effect^32,33^ where the magnetic field causes bulk charge carriers to avoid boundary scattering. In the surface, the scattering conditions are reminiscent of the case of high-mobility bands in a 2D electron gas field effect transistor^34^, a heterostructure where impurity scattering is suppressed because the dopants are separated from the charge carriers by a nanometre-thick spacer. We can check whether this scattering scenario is reasonable for surface states in bismuth nanowires by roughly estimating the change in the orbit diameter *δd*, given a Lorentz force of magnitude ${F}_{{\rm{L}}}=e{V}_{{\rm{F}}}B$, where *V*_F_ = *hk*_F_ /*m*_Σ_ is the Fermi velocity. For a surface-confining potential well^31^, we use a harmonic potential given by (1/2)*k*(*r* –*a*)^2^, where *r* is the radius and *a* ~ *d/2*. The constant *k* is estimated by considering *E*_F_ = *k w* ^2^. A schematic representation of this potential is shown in Fig. 3. Because the change in orbit diameter *δd* = *F*_L_ */k*, we found that *δd* = 1 nm for 10 T. This *δd* is comparable to the width *w* of the interface and therefore, this estimate supports our interpretation of the observations in which Lorentz forces decouple the surface charges from surface scattering.

In the present study, we argue that the observed order of magnitude increase of *G* with *B* is a consequence of the increasing number of high-mobility spiral modes. Another effect that could potentially cause the increase in *G* is an increase in the Fermi energy, which in turn would entail an increase in Σ as we increase the field. We ruled out this mechanism by performing the measurements of *α* that together with the conductance can enable an estimate of the steady, non-oscillatory part *E*_F_(*B*). Applying the Mott relation^35,36^ to the amplitudes, we found (see Supplementary Figure 10) that *E*_F_ increases by approximately 7 meV for 10 T from *E*_F_ ~ 7.6 meV (SdH) or 76 meV (ARPES) without a magnetic field. This result would correspond to a modest 30% increase in Σ in the less favourable scenario (SdH), and, therefore, increases in the Fermi level are too small to explain the observed factor of 10 increase in *G*. The Mott relation also explains the presence of a strong oscillatory component in *α*, as observed in Fig. 2. The Supplementary Material presents our study of coherence length based on the observation of weak antilocalization for small transverse magnetic fields and zero longitudinal magnetic field. We find coherence lengths *L*_ϕ_ of 880 nm and 600 nm at 1.4 K and 2.8 K, respectively. Accordingly, *L*_ϕ_≫ C, where C = π*d* is the circumference. Therefore, it is not reasonable to expect Altshuler-Aronov-Spivak (AAS) *h*/2*e* periods in our nanowires.

There is a strong contrast between the spiral modes of Bi and the helical modes of TIs. The dispersion relation of TIs is Dirac-like, linear with the momentum, and with spin locking, whereas the modes that we discuss here are based on spin-split parabolic bands. The sub-bands in TIs undergo periodic *B*-induced topological transitions. In Bi, the involvement of such phenomena was ruled out in favour of spiral modes since, characteristically, TI with helical modes have not demonstrated steps with net increase in *G*.

In conclusion, we find that 50-nm Bi nanowires exhibit quantized (stepwise) electronic transport under an applied magnetic field and present experimental evidence that surface bands exhibit very long mean free paths and low-temperature high mobilities exceeding 130,000 cm^2^V^−1^s^−1^ and likely reaching 410,000 cm^2^V^−1^s^−1^, comparable (within an order of magnitude) to those observed in suspended graphene. Our surface model, that involves a surface well, is in contrast to the sharp model previously assumed for surface states. In this new model, the surface states scattering conditions are reminiscent of those present in high-mobility 2D electron gases. High mobility is only observed when there is a space between the charges and the surface. This is consistent with lack of backscattering suppression which is in turn consistent with the character of bismuth which is not a topological insulator. The surface spiral modes properties, quantum behavior, high mobility, and underlying topological nature, can be exploited in nanoscale spintronics and thermoelectric applications.

## Methods

We fabricated 50-nm nanowires by applying Taylor’s method, which involved stretching a wire to reduce its diameter. We started with fibres containing large-diameter (~200-nm) nanowires that can be fabricated using the Ulitovsky method. The bismuth is 99.999% pure. See Supplementary Figure 1. The fibres were stretched in a micropipette puller. In a microwire puller, a short section (a fraction of a millimetre in length) was brought to its softening point through the use of a coiled electrical filament that was coaxial with the fibre. When the softening point of the capillary tubing (~500 °C) was reached, a mechanical pulling force was applied to each end of the fibre; the fibre deformed, and the heated section elongated and became thinner. Because the fibre contained a Bi filament, the action of the micropipette puller was to reduce the diameter of the glass fibre and the Bi nanowire simultaneously. In the third (and final) step of fabrication, the nanowires were placed in a travelling heater/oven in which a narrow region of a wire was heated, and this zone was moved slowly along the wire. Keeping in mind that Bi is easily oxidized in contact with air, it is expected that encapsulation of the Bi filament in the glass fibre protected our nanowire samples from oxidation. Electrical connections to the nanowires were performed using a Ga_0.5_In_0.5_ eutectic. The electrical and thermoelectric measurements were performed at the International Laboratory of High Magnetic Fields and Low Temperatures (ILHMFLT), Wroclaw, Poland. The circuit arrangement is presented in Supplementary Figure 3 and the sample rotator is presented in Supplementary Figure 4. Crystalline orientation is depicted in Supplementary Figure 6.

## Electronic supplementary material

Supplementary Information
