# Supplementary material for: Spiral Modes and the Observation of Quantized Conductance in the Surface Bands of Bismuth Nanowires
Source: Sci Rep. 2017 Nov 14;7:15569. doi: 10.1038/s41598-017-15476-5 (PMC5686132; doi:10.1038/s41598-017-15476-5)
Supplement: Supplementary file 1 — Supplementary Information [file 41598_2017_15476_MOESM1_ESM.pdf]

Spiral Modes and the Observation of Quantized Conductance in the Surface Bands of Bismuth Nanowires

Supplementary Information

T. E. Huber,[1*] S. Johnson,[1] L. Konopko,[2,3] A. Nikolaeva,[2,3] A. Kobylianskaya[2] and M. J. Graf [4]

[*]*Correspondence to T.E.H: thuber@howard.edu*

[1] Howard University, Washington, DC 20059, USA

[2] Academy of Sciences, Chisinau, Moldova, MD-2028

[3] International Laboratory of High Magnetic Fields and Low Temperatures, 53-421 Wroclaw, Poland

[4] Department of Physics, Boston College, Chestnut Hill, MA 02467, USA

Table of contents:

S1. Fabrication and Characterization of 45- to 55-nm Nanowires

S2. Electronic Transport: Resistance and Thermopower Methods

S3. 50-nm Wire Anisotropy

S4. SdH Oscillations of Surface Origin

S5. Fermi Energy versus Longitudinal Magnetic Field

S6. UCF, WAL and Coherence Length Study

S7. Supplementary References

S8. Supplementary Figures

S1. Fabrication and characterization of 45- to 55-nm Bi nanowires

For this work, samples of long individual Bi nanowires were fabricated using several steps. The samples that are used in the present investigation are named Q_0, Q_1, Q_2, Q_3 and Q_4. In the first step, Bi wires with a diameter of 200 nm were prepared using a special glass fibre drawing process, also referred to as the Ulitovsky technique[1], by which a high-frequency induction coil melts a 99.999% pure Bi boule within a borosilicate glass capsule, simultaneously softening the glass. The capillary glass has a softening temperature of approximately 500°C. Glass fibres (length is measured in the tens of centimetres) in the form of capillaries containing Bi nanowires were produced by drawing material from the glass capsule. The Ulitovsky method is illustrated in Supplementary Fig. 1. Typically, Ulitovsky Bi wires have diameters ranging between 200 nm and 1 μm; each glass fibre is 10 μm in diameter. Their characterization has been discussed previously[2]. Supplementary Fig. 2 shows a scanning electron microscopy (SEM) image of the cross-section of a Bi wire in its glass fibre.

Our goal was to investigate nanowires with diameters of about 50 nm. We were unsuccessful in our attempts to use the Ulitovsky technique to produce wires with a diameter of less than 200 nm. However, we were successful in making 50-nm nanowires by applying Taylor's method[3], a widely used method that involves stretching a wire to reduce its diameter. We placed fibres containing large-diameter nanowires (~200 nm), in a micropipette puller. Our puller is homemade and is a refinement of the one manufactured by Warner Instruments (1125 Dixwell Avenue, Hamden, CT 06514, USA). In a puller, a short section (a fraction of a millimetre in length) of the fibre was brought to its softening point through the use of a coiled electrical filament that was coaxial with the fibre. When the softening point of the capillary tubing

(~500°C) was reached, a mechanical pulling force was applied to each end of the fibre; the fibre deformed, and the heated section elongated and became thinner. Because this deformation can evolve into a neck, which in turn evolves into a break or discontinuity, the process was controlled with an electronic controller loaded with a programme that considers variables such as capillary material type, temperature, heating filament type and pulling force[4]. Clearly, since the fibre contained a Bi filament, the action of the micropipette puller was to reduce the diameters of the glass fibre and the Bi nanowire simultaneously. In the third (and final) step of fabrication, the fibres were placed in a travelling heater/oven, where a narrow zone of a fibre is heated, and this zone is made to be moved slowly along the fibre. The travelling heater technique was employed to anneal and prepare homogenous single crystals of BiSb alloys successfully[5]. When in contact in air, Bi is easily oxidized; therefore we believe that the glass fibre protects our nanowire samples from oxidation. Because Bi has a bright metallic shine inside the glass fibre, Bi nanowires with a diameter as small as 50 nm are visible with an optical microscope under plain microscope settings. Therefore, it is possible to select sections of fibre that are roughly 0.5 mm in length and that are homogenous in brightness along the length. Such sections are suitable for transport measurements. The assumption is that optical scattering is a strong function of the diameter. Thus, homogenous optical scattering is an indicator of a constant diameter. Our research is based on these uniform (constant diameter) sections. Several batches of samples of wires with diameters between 55 nm and 45 nm were studied. Samples are characterized by an electronic diameter $d = (4l/\pi\sigma(300\text{ K})R(300\text{ K}))^{1/2}$, where l is the wire length; $\sigma(300\text{ K})$ is the Bi room temperature conductivity of bulk Bi, which is $8.7\times10^3\ \Omega^{-1}\text{cm}^{-1}$; and $R(300\text{ K})$ is the wire resistance. Because length l is short and cannot be measured well, this leads to a 20% error in the electronic diameter. Figure 1 of our paper shows an SEM image of a sample named Q1, which has an electronic diameter of 50 nm. In Supplementary Figure. 2, we show an SEM image, taken with a high resolution JEOL microscope (Materials Research Science and Engineering Center at Johns Hopkins University), of a nanowire sample named Q2, which has an electronic diameter of

55 nm. The measurement of the diameter on the SEM image is 70 nm, with an error of 5%. Therefore, a discrepancy of 15 nm exists between the diameter obtained with SEM and the electronic diameter. This discrepancy might be explained by the error in the electronic diameter. It is also important to mention the poor conditions we encountered for SEM imaging. We were unable to focus on the Bi interface because of glass charging, and, therefore, it is possible that the SEM images are affected.

S2. Nanowire electronic transport: resistance and thermopower experimental methods

Electrical connections to the nanowires were performed using Ga(0.5)In(0.5) eutectic (GaIn). The special properties of GaIn as a room temperature solder are well known[6]. In our experience, this type of solder consistently makes good contacts, but it has the disadvantage that some of the alloy constituents diffuse at room temperature into the nanowire. The nanowires were used in the low-temperature experiment within ~1 h after application of the solder; otherwise, the oscillations of the magnetoresistance that we have discussed are not observed. For our work, we measured, for the Q1 sample, the low temperature thermopower α simultaneously with the resistance and magnetoresistance. These results, in the range of magnetic fields up to 15 T, are shown in Fig. 2 of our paper. Supplementary Figures 3 and 4 illustrate the experimental setup. These devices allowed us to measure resistance directly, without using a four-contact configuration, because the wires were long and the contact resistance was small in comparison. Figure 3 of our paper confirms that the contact resistance is negligible by showing that the resistance decreased by an order of magnitude between B = 0 and 15 T. Accordingly, we estimated the contact resistance to be only 25 KΩ. The heater allowed us to create a temperature difference between the two electrodes and, therefore, between the two ends of the wires. The thermopower α value was estimated using $\alpha = \Delta V/\Delta T$, where ΔV is the electric potential generated across the nanowire in response to temperature difference ΔT between the nanowire

ends. The measurements were performed at the International Laboratory of High Magnetic Fields and Low Temperatures (ILHMFLT), Wroclaw, Poland.

The temperature-dependent resistance of a 45-nm wire, sample Q3, is shown in Supplementary Figure 5. We found $R(T) \sim \exp(-(\Delta/T))$ for 30 K < T < 100 K, where Δ, which can be interpreted as the semiconductor gap between the electron and hole band in the core of the nanowires, is 14 meV. This value of the gap is in agreement with theoretical work indicating that the band overlap decreases substantially below the value for bulk Bi (38 meV) owing to quantum confinement. Therefore, one expects the charge carrier density in our nanowires to be less than n (the bulk electron and hole densities). The nanowire resistance becomes saturated at T < 30 K, owing to surface electronic transport. It can be surmised that the observed low-temperature transport, which caused the saturation of $R(T)$ at low temperature, was mediated by charges in surface states. The sheet resistance R_s of surface states was found to be around 590 Ω/sq.

The X-ray scattering of a bundle of Ulitovsky nanowires is strong enough that it can be used for crystalline structural determinations[2]. Ulitovsky nanowires also display Shubnikov-de Haas (SdH) oscillations of the magnetoresistance associated with the Fermi surface of bulk Bi in nanowires[8,9]. Both X-ray studies on the crystalline structure and studies on the Fermi surface have shown that nanowires grown by the Ulitovsky method are single-crystal nanowires and that the orientation of the crystalline structure is such that the wire axis is in the bisectrix-trigonal plane, making an angle of ~70° with the trigonal axis (C3). This is comparable to the orientation reported by Heremans *et al*.[10] for Bi nanowires of various diameters synthesized by vapour phase injection of porous alumina templates. Supplementary Figure 6 illustrates the typical orientation of Ulitovsky nanowires with respect to the Fermi surface of bulk electrons and holes.

S3. 50-nm wire anisotropy

The crystalline structure of the large-diameter nanowires (larger than about 200 nm) was investigated with the traditional methods of X-ray diffraction, transmission electron microscopy (TEM) and SdH oscillations. In contrast to large-diameter nanowires, the small diameter of the 50-nm nanowires makes it difficult to employ X-ray diffraction; for this reason, we were not able to determine the structure with this method. In addition, TEM did not work because the wire was immersed in glass. The wires also did not exhibit SdH oscillations, periodic in $1/B$ that can be related to the bulk Fermi surface; this lack of SdH oscillations is expected because Bi is a bulk insulator owing to quantum confinement. Therefore, using traditional methods to determine the crystalline structure is a difficult task. However, we observed the nanowire electrical transport, and, considering that bismuth is very anisotropic, we looked for measurements of the anisotropy that would have provided evidence of the crystalline structure. Bulk-like states obey crystal symmetry; therefore, one can expect surface states to obey symmetry also. In wires, transverse magnetoresistance (TMR) is the magnetoresistance (MR) with the magnetic field perpendicular to the wire axis. We observed the TMR to be strongly dependent on the orientation of the nanowire specimen around the nanowire axis; that is, the TMR is anisotropic. If angle β refers to the relative orientation of the nanowire specimen around the nanowire axis with respect to the magnet, then the TMR anisotropy consists of a unique pattern of oscillations of the angle-dependent TMR(β). This anisotropy has been previously shown in samples that we have studied previously and demonstrated h/e and $h/2e$ oscillation[11].

The TMR(β) of large single-crystal specimens of bismuth, doped bismuth and bismuth antimony has been studied experimentally and theoretically[12-14]. The TMR anisotropy reflects the asymmetry of the Fermi surface combined with the asymmetry of the crystal specimen plus contacts. In single-crystal nanowire samples of Bi and BiSb with contacts at the nanowire ends, the TMR(β) was observed to be symmetric around the C1 axis. At low magnetic fields and for large specimens, the anisotropy is explained with Aubrey theory[14], where the anisotropy is attributed to the anisotropy of the conductivity and mobility tensors. The (weak magnetic field)

TMR(β) of the 50-nm nanowire is shown in Supplementary Figure 7. We also show the weak magnetic fields TMR of a large-diameter 330-nm nanowire grown with the Ulitovsky technique[9]. The 330-nm wire is representative of a large-diameter Bi nanowire. It is a bulk conductor that supports Landau states, as evidenced by the presence of SdH oscillations. Both cases are anisotropic and relatively similar in that they are binary symmetric around an axis.

Our comparison of 330-nm Bi nanowires and 45-nm Bi nanowires yielded rather interesting findings. We found the 330-nm wire to be a bulk conductor and no evidence of surface states. Therefore, the observed anisotropy can be accounted for by the shape of the Fermi surface, where the Fermi surface in the nanowire is almost the same as in the bulk conductor. The saddle point that is observed for $\beta = 0$, which corresponds to the orientation where B is aligned with the binary crystalline axis, is a distinguishing feature. The TMR of bulk Bi crystals exhibit the same TMR saddle point in the binary direction[12]. By contrast, small-diameter nanowires that exhibit surface electronic transport and are bulk insulators—to the extent that they exhibit AB oscillations but not SdH oscillations—also exhibit binary anisotropy. This can be understood only if the small-diameter nanowires are single-crystal nanowires and have the same binary anisotropy as the large-diameter nanowires. Clearly, this anisotropy cannot be derived from the anisotropy of the Fermi surface, that is, electrons and hole pockets, because Bi is a bulk insulator. Rather, the binary anisotropy of the 50-nm nanowires must be related to the properties of surface states. Preliminarily, we have been able to explain the anisotropy of the TMR in Supplementary Figure 7 by considering that the surface is imprinted with (111) edges so that the electron gas at the surface then has to satisfy geometrical constraints and, in particular, the binary symmetry of the lattice. This model is especially relevant because it *also* explains the presence and behaviour of the anisotropy of the AB oscillations for small-diameter Bi nanowires when the field is perpendicular to the wire axis and there is apparently no flux through the nanowire. Konopko *et al.*[7] argued that the path followed by the charge carriers on the surface is not in circles that are perpendicular to the wire axis; rather, the path followed by the charge carriers on

the surface is in circles that follow the edge of the (111) bilayers because this path offers less resistance.

S4. SdH oscillations of surface origin

We observed SdH oscillations (periodic in $1/B$) of the TMR, that is, the magnetoresistance with the field perpendicular to the wire axis at low temperatures. We considered the presence of AB oscillations to be strong evidence of the two dimensional (2D) character of the surface, and, therefore, we assigned the SdH oscillations to Landau states in the surface. SdH oscillations correspond to successive emptying of Landau levels (LL) as the magnetic field increases. The relationship of the LL index to the extremal cross-section S_F of the Fermi surface (FS) was calculated using the following:

$$2\pi(n+\gamma) = S_F\, h/(2\pi eB)\,, \qquad (1)$$

where e is the electron charge, h is Planck's constant and B is the magnetic flux density. Assuming an isotropic 2D gas and disregarding the curvature of the nanowire surface, $S_F = \pi k_F$. Because γ is related to spin dynamics, it is also related to the topological character of the surface. The SdH period, the surface-state charge and the topological character of the surface are discussed in our paper. Here we analyse the temperature and magnetic field dependence of the SdH amplitudes of sample Q_4, also called A96 in our laboratory notebooks. We also discuss certain aspects of the analysis so as to obtain information about the effective mass and transport lifetime of the surface states. Our analysis is based on the theory of SdH oscillations developed by Lifshitz and Kosevich[15]. This approach was tested in bulk (three-dimensional) metals and semiconductors by Sladek[16]. Recently, Taskin and Ando[17] applied this method to 2D layers of topological insulators (TIs). As shown in Supplementary Fig. 8(b), the oscillation amplitude in dR/dB decreases rapidly as T is raised from 1.4 K to 2.8 K. In the index plot of Supplementary

Fig. 8(a), we confirmed that the inverse peak fields $1/B$ at the extrema (maxima and minima) fall on a straight line versus the extrema index (integer n) (equation [1]). The figure shows that the simple linear fit gives an intercept of 0.35 an apparently non-trivial value that is unexpected because theoretical analysis indicates that bulk Bi has a trivial TI character[18,19], for which the Berry phase is zero. When we modelled the SdH points with a non-linear fit that includes spin-orbit coupling (SOC)[20], with a g-factor of 20, we obtained the expected result; that is, the intercept, and therefore the Berry phase, is zero. However, we were not able to cross-check the value of g independently. The charge density per unit area Σ was estimated from the SdH period (P = 0.060 T^{-1}) using $\Sigma = f/(P\Phi_0)$, where f is the 2D Landau level degeneracy, which is two on account of the two-fold spin degeneracy. We found $\Sigma = 8.06 \times 10^{11}/\mathrm{cm}^2$, which was an order of magnitude smaller that the ARPES measurement[21] of $8 \times 10^{12}/\mathrm{cm}^2$ for Bi crystals. The 2D Fermi energy $E_F = \pi h^2 h^2 \Sigma / m_\Sigma$ and k_F were found to be 7.6 meV and 2.2×10^8/m, respectively. Taking Σ to be $8 \times 10^{12}/\mathrm{cm}^2$ (ARPES value), we estimated E_F = 76 meV and $k_F = 7.1 \times 10^8$/m. Below, we will argue that the values from ARPES is considered more appropriate for our nanowires than the values that are derived from the SdH measurements.

Because, in the last paragraph, success was achieved in fitting positions of the maxima and minima of the dR/dB to a sequence of Landau levels, we next attempted to fit the amplitudes with the theory[15-17]. We cannot claim the same degree of success. The T dependence of the amplitude of magnetoresistance oscillations is given using the following:

$$\Delta R / R_0 \sim \lambda(T) / \sinh \lambda(T) \exp(-D), \qquad (2)$$

where R_0 is the resistance, ΔR is the amplitude of the oscillation of the resistance that is assigned to SdH, and $\lambda(T) = \pi^2 k_B T / \hbar\omega_c$ is the thermal factor where k_B is the Boltzmann constant and D is the Dingle factor[22,23]. Here $\hbar\omega_c = 2\mu_B B / m_\Sigma$. The cyclotron mass of the surface states is m_Σ. ΔR is obtained by integration of dR/dB after subtraction of a smooth background. This equation for

the amplitudes is the same recently used by Qu *et al.*[24] in their analysis of magnetoresistance MR data. $D = -2\pi^2 k_B T_D/\hbar\omega_c$ and T_D is the Dingle temperature. We found that the oscillation amplitudes depend on B and T. This is shown in Supplementary Figure 9. However, the data could not be fit with Equation 2 except for the highest magnetic field points.

The effective mass was obtained using the magnetoresistance for 1.4 K and 2.8 K by applying the following:

$$m_\Sigma = \frac{\mu_B B_n}{4\pi^2 k_\mathbf{B} T_1} \text{arcosh}[\text{MR}(\text{T}_1,\text{B}_\text{n})/\text{MR}(2\text{T}_1,\text{B}_\text{n})], \qquad (3)$$

where T_1 is 1.4 K and B_n corresponds to the maxima of derivative. We found $m_\Sigma = 0.25 \pm 0.05$. We also found T_D = 4.9 K. The semiclassical transport collision time τ is $\tau > \tau_D = \hbar/k_B T_D = 3 \times 10^{-12}$ s. The mobility $\mu > e\tau_D/m_\Sigma m_e$. The mobility estimate, which is a lower limit because the actual relaxation time τ is longer than the broadening embedded in τ_D, is 12,000 $cm^2\, s^{-1}\, V^{-1}$. This mobility is of the same order of magnitude as the one derived by Qu *et al.*[24] for the surface states of Bi_2Te_3 using similar methods. Our values for mobility also compare favourably with those reported by Ning *et al.*[25] This value of mobility that we derived from SdH analysis for the surface states of Bi nanowires is at least one order of magnitude less than the values derived for the 50-nm nanowires in our paper, which we determined through analysis of the stepwise conductance or from the direct measurement of the conductance. We found that the low-temperature mobilities exceeded 130,000 $cm^2V^{-1}s^{-1}$ and likely reached 410,000 $cm^2V^{-1}s^{-1}$ at low temperatures, comparable (within an order of magnitude) to those observed in suspended graphene.

The analysis of the amplitude of SdH oscillations is not straightforward because of the cylindrical geometry of the wires. Treating this as a flat surface that is perpendicular to the field is not obviously valid for the cylindrical geometry of a nanowire because most of the surface is not perpendicular to the magnetic field, even if the field is perpendicular to the wire axis.

If we consider a nanowire in a transverse magnetic field (field perpendicular to the nanowire axis), it is advantageous to divide the nanowire circumference in four sectors. There are two sectors of the nanowire circumference that are perpendicular to the field. Such a case is illustrated in the inset of Supplementary Figure 8. These sectors represent half of the area of the nanowire and are populated with Landau levels. We will discuss these sectors in the next paragraph. The sectors that are tangential to the magnetic field are discussed in the last paragraph.

Regarding the sectors of the nanowire that are perpendicular to the field, their magnetoresistance is high. Obviously, these sectors are responsible for the observation of SdH phenomena. However, we cannot fully disregard the curvature of the cylinder because the Larmor radius $R_L = hk_F/(2\pi eB)$, which is the semi-classical radius of the orbit of a charged particle in the presence of a uniform magnetic field, is not small compared to the wire diameter. For our SdH values of k_F = 2.2 10^8/m, we obtain R_L = 18 nm for B = 8 T and 36 nm for B = 4 T. If the charge density is 10 times larger, as in the ARPES values, then k_F is three times larger. Then R_L= 56 nm for 8 T and 108 nm for 4 T. Such values of R_L are not negligible in comparison to the diameter. For this reason, we believe that the SdH determination of the charge density based on the SdH data is not correct. Therefore, the value Σ from ARPES is considered more appropriate for our nanowires than the values that are derived from the SdH measurements.

There are sectors that are tangential to the field and represent the other half of the nanowire area. These sectors do not support Landau levels. Their magnetoresistance can be expected to be small. Therefore, in a calculation of the resistance where one considers the resistance in parallel of the sectors that are tangential to the field and the sectors that are perpendicular to the field, the ones that are tangential are dominant and cannot be disregarded.

S5. Fermi energy versus longitudinal magnetic field

We observed at $B = 0$ that the thermopower α is linear for T up to ~50 K, with positive α indicating that the surface charges are holes. Figure 2 and Supplementary Fig 10 show that in addition to the steady part of the thermopower, there is an oscillatory component. The maxima/minima of thermopower correspond to the minima/maxima of dG/dB, indicating thermopower conditions for which there is no phonon drag and the thermopower is diffusive[26]. Furthermore, in the diffusive case, a quantitative correlation between α and G can be made using the Mott relation:

$$\alpha = -\frac{\pi^2 k_B{}^2 T}{3e} \frac{dG/dE_F}{G} \qquad (4)$$

The Mott relation, obtained from the Boltzmann equation in the relaxation-time approximation[27] has been tested experimentally in various materials including semiconductors [26], nanotubes[28], graphene[29] and nanowires[30]. The Mott equation explains the oscillatory thermopower. It should be noted that the thermopower is positive at most magnetic fields but it has negative values is the 2T to 4 T range. The strength of the oscillatory term, that overcomes the steady part of the thermopower, is directly related to our main result that the mobility is high and stepwise. The technique of tuning the carrier density by electric field effect of a gate is particularly useful for examining the Mott relation, since it allows a quantitative comparison between thermopower and conductivity. In this case, G, α and E_F are functions of the gate electric potential. In our experiment there is no gate but an applied magnetic field. In our case, we can write:

$$\alpha = -\frac{\pi^2 k_B{}^2 T}{3e} \frac{\mathrm{d}G/\mathrm{d}B \; dB/dE_F}{G} \qquad (5)$$

Therefore, we find:

$$dE_F/dB = -\frac{\pi^2 k_B{}^2 T}{3e} \frac{dG/dB}{G\alpha} \qquad (6)$$

Consequently, dE_F/dB can be found using the experimental values of $G(B)$ and $\alpha(B)$, specifically the amplitude of the oscillation of the dG/dB and thermopower. shown in Figure 2 of our paper. We derived dE_F/dB from the oscillation amplitude $\Delta(\frac{dG/dB}{G})$ and the oscillation amplitude $\Delta(\alpha)$ The outcome of this procedure for applying the Mott relation to the oscillation amplitudes is shown in Supplementary Fig. 10. We found that E_F increased by approximately 7 meV for 10 T. In the worst-case scenario that E_F = 7.6 meV, this increase would result in a modest 30% increase in Σ, which could not explain the observed 10-fold increase in G in the same range of magnetic fields. The derivation in Equations 4-6 do not depend on defining $G = \mu\Sigma$. However, if we assume that the changes in Σ are very modest, as in our case, the Mott relation leads to the strong oscillatory component of the thermopower as a derivative of the large mobility. This explains the negative thermopower at B = 3 T and 4 T.

S6. UCF, WAL, and Coherence Length Study

We studied the transverse magnetoresistance of nanowire sample Q4. This is the same sample that was studied in our study of SdH oscillations. By performing this measurements at various temperatures, namely 1.4 K and 2.8 K, it was possible to clarify UCF (Universal conductance fluctuations), WAL (weak antilocalization) and to obtain the phase coherence length L_ϕ . Supplementary Figure 11 shows the magnetoresistance as a function of magnetic field, normalized to the resistance at zero magnetic field, for nanowire Q4. The conductance fluctuations (UCF) are not present. The fluctuations that were observed were plain noise. The root mean square (rms) of this noise is less than 10^{-5} e^2/h . This can be understood because UCF correlates with the expression[31] $\delta G \propto (e^2/h)(L_\phi/l)^{3/2}$, where L_ϕ is the coherence length and l is

the channel length that is the length of the nanowire. Since l is 300 μm and it can be expected that the length L_ϕ is hundreds of nm. Therefore, $\delta G/(e^2/h)$ is small. It is easy to distinguish the fluctuations of conductance that we observe with true UCF because they are not symmetric with magnetic field and do not repeat from one measurement to another.

The weak antilocalization effect (WAL) is apparent in the sharp "valley" feature consistent of a decrease of the magnetoresistance $R(B)/R(B = 0 \text{ T})$ shown in Supplemental Figure 11. The breadth of the WAL peak is indicative of the coherence length L_ϕ . It is common to apply the Hikami-Larkin-Nagaoka (HLN) theory to surface states in nanowires. Then the WAL magnetoresistance breadth is $B_\phi = \Phi_0 /4L_\phi$ where $\Phi_0 = h/e$.

$$\delta G_{WAL}(B) = -\alpha \frac{e^2}{2\pi^2\hbar}\left[\psi\left(\frac{1}{2} + B_\phi/B\right) - \ln\left(\frac{B_\phi}{B}\right)\right] \tag{7}$$

where α and ψ are the fitting parameter and the digamma function, respectively. Fitting the above equation to the experimental curves of the MR, see Supplementary Figure 11 and 12, we can then find B_ϕ and therefore L_ϕ. We found the peak breaths to be small and we found long correlation lengths as a result. For example, for 1.4 K, we found L_ϕ= 880 nm that is 16 times the wire diameter. At 2.8 K, L_ϕ= 600 nm. In comparison, the study by Ning *et al*, Supplementary Reference 25 gives results ranging 60 and 300 nm at low temperatures. Clearly this equation is supposed to be applied to (flat, infinite) disordered two-dimensional systems and therefore its use in nanowires that have little disorder, of very small diameters, is only tentative.

S7. Supplementary References

1. Ulitovsky, A. V. in *Microtechnology in the Design of Electrical Devices,* Vol. 7, p. 6 (Leningrad, 1951).

2. Nikolaeva, A., Huber, T. E., Gitsu, D. & Konopko, L. D. Diameter-dependent thermopower of bismuth nanowires. *Phys. Rev. B* **77**, 035422 (2008).

3. Taylor, G. F. A method of drawing metallic filaments and a discussion of their properties and uses. *Phys. Rev.* **23**, 655–660 (1924).

4. Brown, K. T. & Flaming, D. G. *Advanced Micropipette Techniques for Cell Physiology* (John Wiley and Sons, New York, 1986).

5. Lenoir, B. *et al.* Growth of $Bi_{1-x}Sb_x$ alloys by the traveling heater method. *J. Phys. Chem. Solids* **56**, 99–105 (1995).

6. Dickey, M. D. *et al*. Eutectic gallium-indium (EGaIn): a liquid metal alloy for the formation of stable structures in microchannels at room temperature. *Adv. Funct. Mater.* **18**, 1097–1104 (2008).

7. Konopko, L. A., Huber, T. E., Nikolaeva, A. A. & Burceacov, L. A. Quantum interference of surface states in bismuth nanowires in transverse magnetic fields. *J. Low Temp. Phys.* **171**, 677–684 (2013).

8. Nikolaeva, A., Huber, T. E., Konopko, L. & Tsurkan, A. Observation of the semiconductor-semimetal and semimetal-semiconductor transitions in Bi quantum wires induced by anisotropic deformation and magnetic field. *J. Low Temp. Phys.* **158**, 530–535 (2010).

9. Gitsu, D., Konopko, L., Nikolaeva, A. & Huber, T. E. Pressure-dependent thermopower of individual Bi nanowires. *Appl. Phys. Lett.* **86**, 102105 (2005).

10. Heremans, J. *et al.* Bismuth nanowire arrays: synthesis and galvanomagnetic properties. *Phys. Rev. B* **61**, 2921–2930 (2000).

11. Nikolaeva, A., Gitsu, D., Konopko, L., Graf, M. J. & Huber, T. E. Quantum interference of surface states in bismuth nanowires probed by the Aharonov-Bohm oscillatory behavior of the magnetoresistance. *Phys. Rev. B* **77**, 075332 (2008).

12. Brown, R. D., Hartman, R. L. & Koenig, S. H. Tilt of the electron Fermic surface in Bi. *Phys. Rev.* **172**, 598 (1968).

13. Jacobson, D. M. Magnetoresistance anisotropy in bismuth and bismuth-antimony crystals. *Phys. Status Solidi B* **58**, 243–250 (1973).

14. Aubrey, J. E. Magnetoconductivity tensor for semimetals. *J. Phys. F Met. Phys*. **1**, 493 (1971).

15. Lifshitz, I. M. & Kosevich, A. M. *Zh. Eksp. Teor. Fiz.* **29**, 730 (1955) [*Sov. Phys. JETP* **2**, 636 (1956)].

16. Sladek, R. J. Magnetoresistance oscillations in single-crystal and polycrystalline indium arsenide. *Phys. Rev.* **110**, 817–826 (1958).

17. Taskin A. A. & Ando Y. Quantum oscillations in a topological insulator $Bi_{1-x}Sb_x$. *Phys. Rev. B* **80**, 085303 (2009).

18. Hasan, M. Z. & Kane, C. L. *Colloquium:* Topological insulators. *Rev. Mod. Phys.* **82**, 3045–3067 (2010).

19. Fu, L. & Kane, C. L. Topological insulators with inversion symmetry. *Phys. Rev. B* **76**, 045302 (2007).

20. Taskin, A. A. & Ando, Y. Berry phase of nonideal Dirac fermions in topological insulators. *Phys. Rev. B* **84**, 035301 (2011).

21. Ast, C. R. & Höchst, H. Fermi surface of Bi(111) measured by photoemission spectroscopy. *Phys. Rev. Lett*. **87**, 177602 (2001).

22. Dingle, R. B. Some magnetic properties of metals. I. General introduction, and properties of large systems of electrons. *Proc. Roy. Soc. Lond. A* **211**, 500–516 (1952).

23. Dingle, R. B. II. The influence of collisions on the magnetic behaviour of large systems. *Proc. Roy. Soc. Lond. A* **211**, 500–516 (1952).

24. Qu, D.-X., Hor, Y. S., Xiong, J., Cava, R. J. & Ong, N. P. Quantum oscillations and Hall anomaly of surface states in the topological insulator Bi_2Te_3. *Science* **329**, 821–824 (2010).

25. Ning, W. *et al.* Evidence of topological two-dimensional metallic surface states in thin bismuth nanoribbons. *ACS Nano* **8,** 7506−7512 (2014).

26. Fletcher, R. Magnetothermoelectric effects in semiconductor systems. *Semicond. Sci. Technol.* **14**, R1–R15 (1999).

27. Ziman, J. M. *Principles of the Theory of Solids* (Cambridge Univ. Press, Cambridge, 1965).

28. Small, J. P. *et al.* Modulation of thermoelectric power of individual carbon nanotubes. *Phys. Rev. Lett*. 91, 256801 (2003).

29. Ghahari F. *et al.* Enhanced thermoelectric power in graphene: Violation of the Mott relation by inelastic scattering. *Phys. Rev. Lett*. 116, 136802 (2016).

30. Tian, Y. *et al*. One-dimensional quantum confinement effect modulated thermoelectric properties in InAs nanowires. *Nano Lett*. 12, 6492-6497 (2012).

31. Ibach, H., and Luth H., *Solid State Physics: An Introduction to Principles of Materials Science* (Springer, New York, 2009), p. 278..

32. Hikami, S. *et al.* Spin-Orbit Interaction and Magnetoresistance in the Two Dimensional Random System. *Prog. Theor. Phys*., 63, 707-710 (1980).

S8. Supplementary Figures

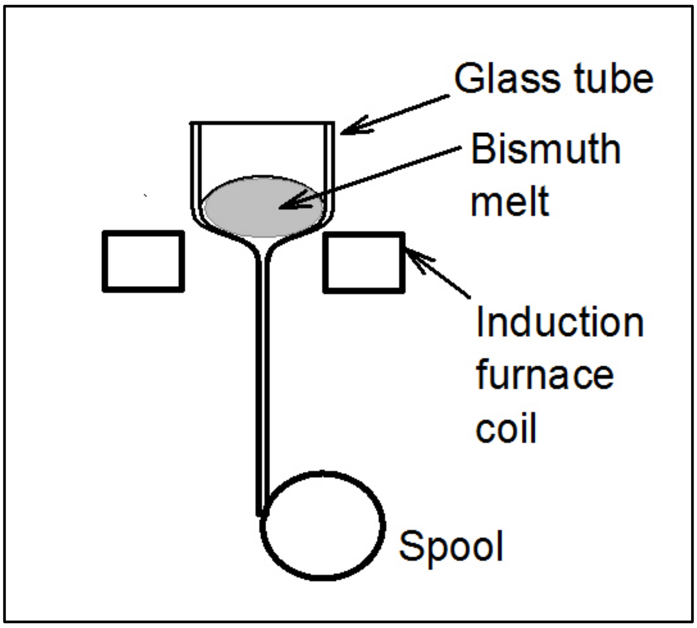

Supplementary Figure 1. Schematic diagram of the Ulitovsky method. The diagram shows the glass fibre that contains the metal (bismuth) nanowire (~200 nm) and the induction coil that heats up the metal. The fibre is retrieved at high speed (metre per second) and collected in a spool.

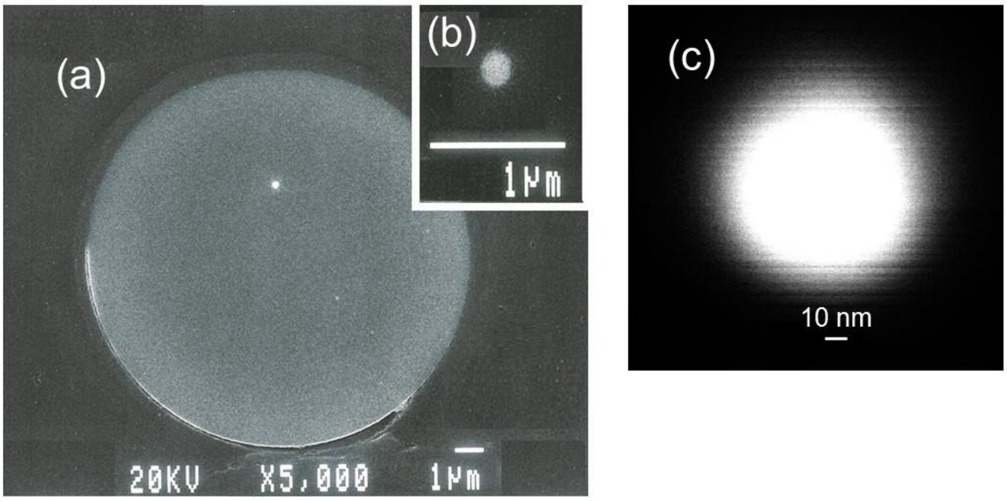

Supplementary Figure 2. Scanning electron microscope. (a) Cross-sectional scanning electron microscopy (SEM) image of a 160-nm wire (clear) in its glass fibre. **(b)** Cross-sectional SEM image of the 160-nm nanowire at higher magnification. **(c)** Cross-sectional SEM image of the 50-nm nanowire named Q2 at higher magnification.

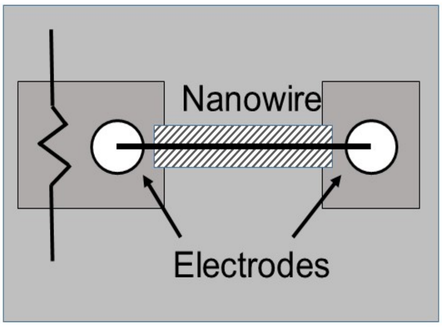

Supplementary Figure 3. Sample resistance and thermopower nanowire circuit arrangement. Schematic of the arrangement used for the electrical resistance and thermopower measurements. A heater establishes a temperature difference between the two sides of the nanowire. The device can be aligned with the magnetic field using a two-axis rotator (see Supplementary Figure 4), which is available at the International Laboratory of High Magnetic Fields and Low Temperatures (ILHMFLT).

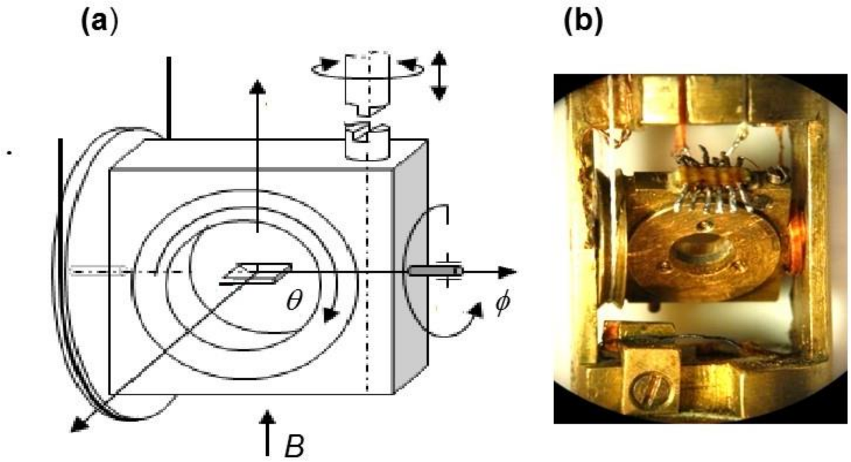

Supplementary Figure 4. Sample rotator. (a) Schematic and **(b)** photograph of the two-axis rotator that we employed in our experiments. The device shown in Supplementary Fig. 3, used for the resistance and thermopower measurements, is mounted in this rotator. In this manner, we can position the nanowire at a controllable orientation with respect to the magnetic field. Angle β measures the orientation of the Bi nanowire around its own axis, whereas angle θ is the angle between the nanowire axis and the magnetic field *B*.

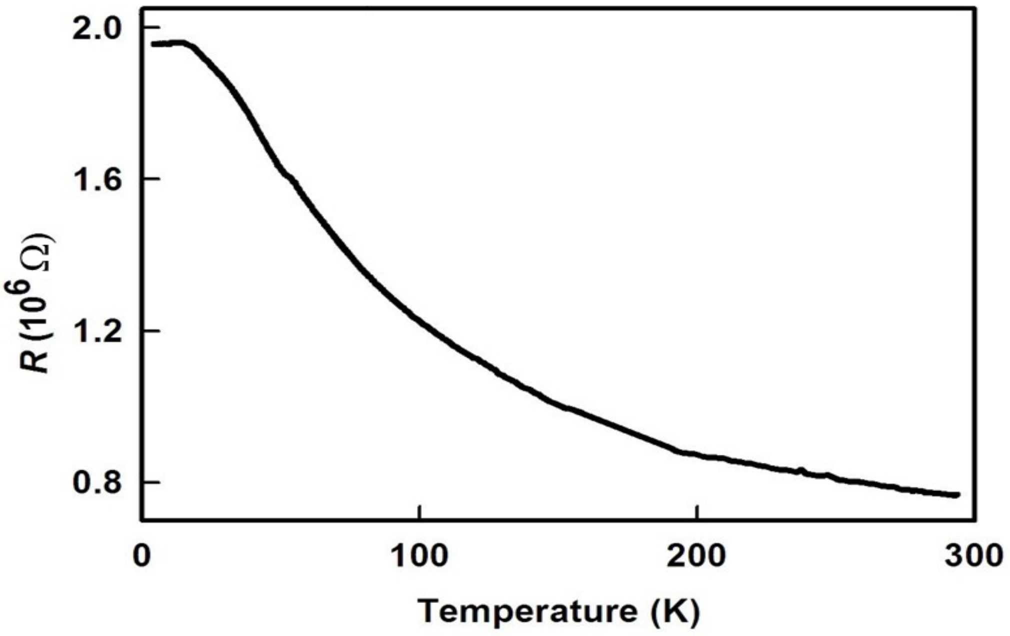

Supplementary Figure 5. Temperature-dependent resistance. The 45-nm nanowire sample named Q3 was studied. The length is 0.5 ± 0.1 mm. Adapted from Ref. 7.

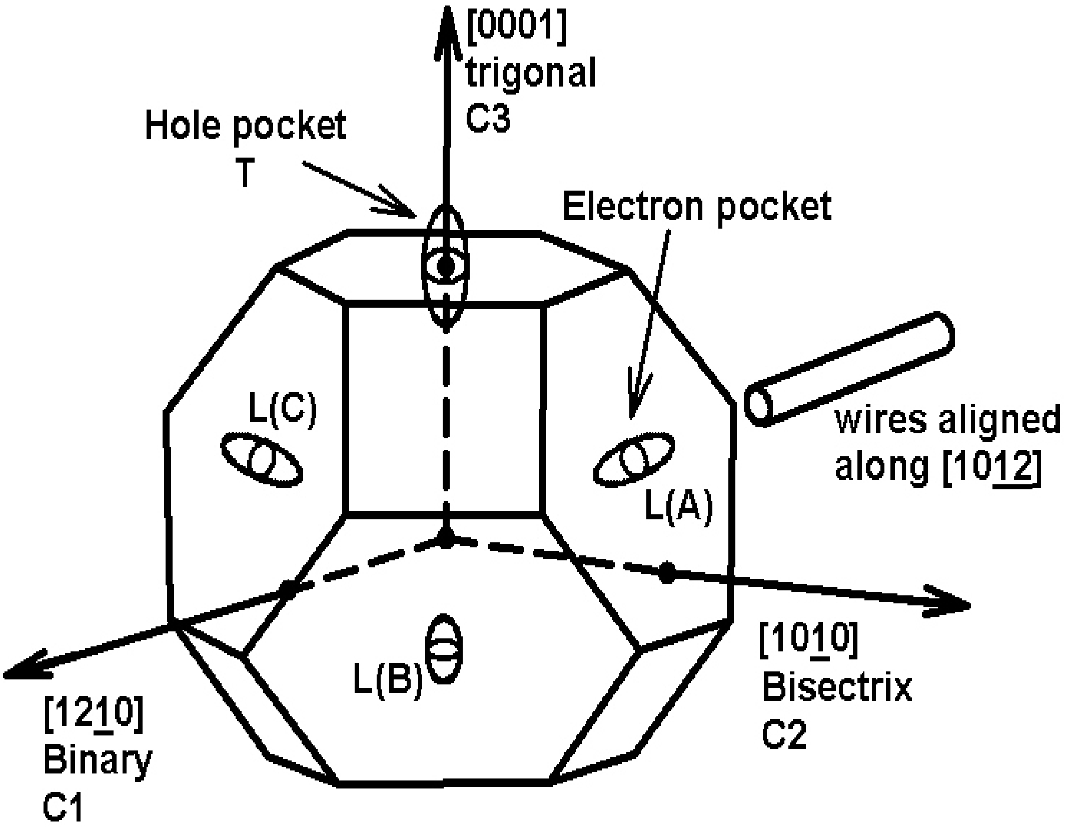

Supplementary Figure 6 . Orientation of the nanowires. The diagram depicts the Brillouin zone for Bi, showing lines of symmetry that are indexed in the hexagonal system, and planes showing the orientation of the wires in the present study. Three Fermi surface electron pockets—L(A), L(B) and L(C)—and a T-point hole pocket are also shown. Three other electron pockets and another hole pocket are symmetric with respect to the centre of the Brillouin zone but are obscured from view. We illustrate the typical orientation of Ulitovsky wires with respect to the Brillouin zone.

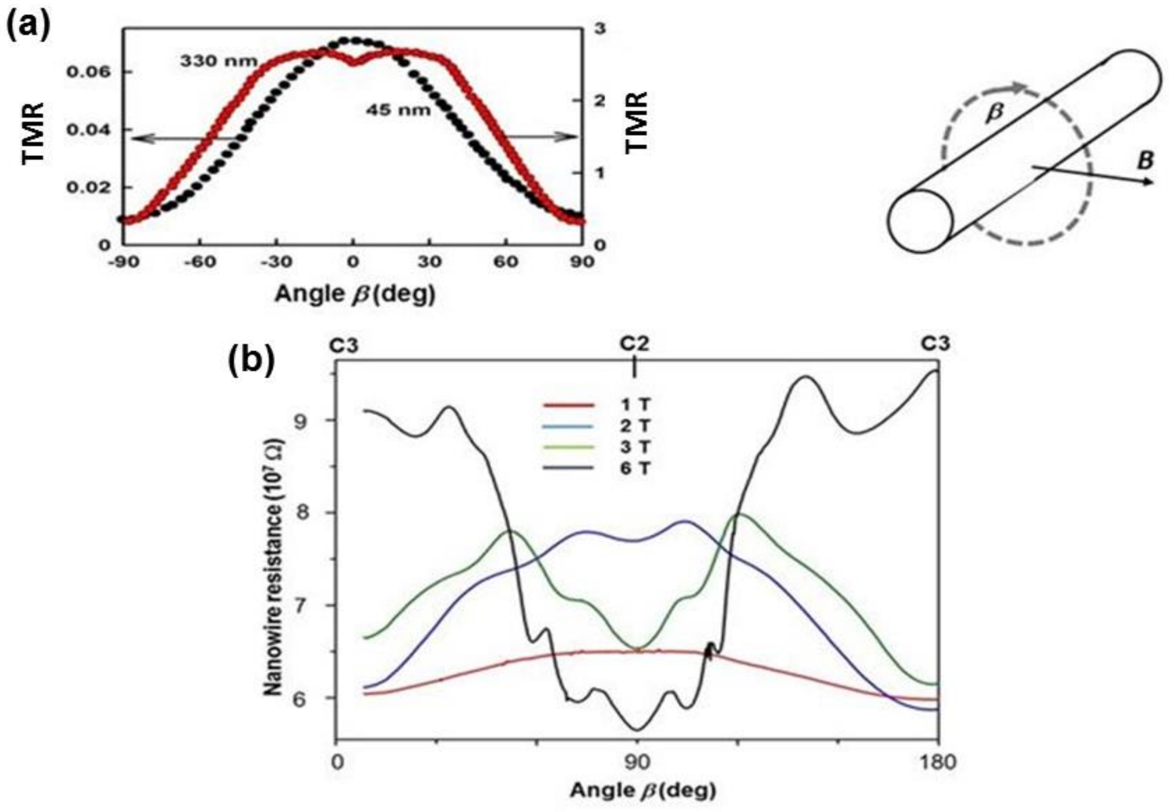

Supplementary Figure 7. Magnetic field transverse magnetoresistance (TMR) rotational diagrams of Bi nanowires. TMR = $R(B)/R(0)$-1 for magnetic fields perpendicular to the wire axis **(a)** Weak magnetic field comparison. The diameters are 45 nm (Q3) and 330 nm. The temperature is 4.2 K, and the field is 1 T. Adapted from Ref. 8. **(b)** TMR of the 45-nm nanowire Q3 for various magnetic fields. We show the presumed orientation of the nanowire with respect to the bisectrix and trigonal axis.

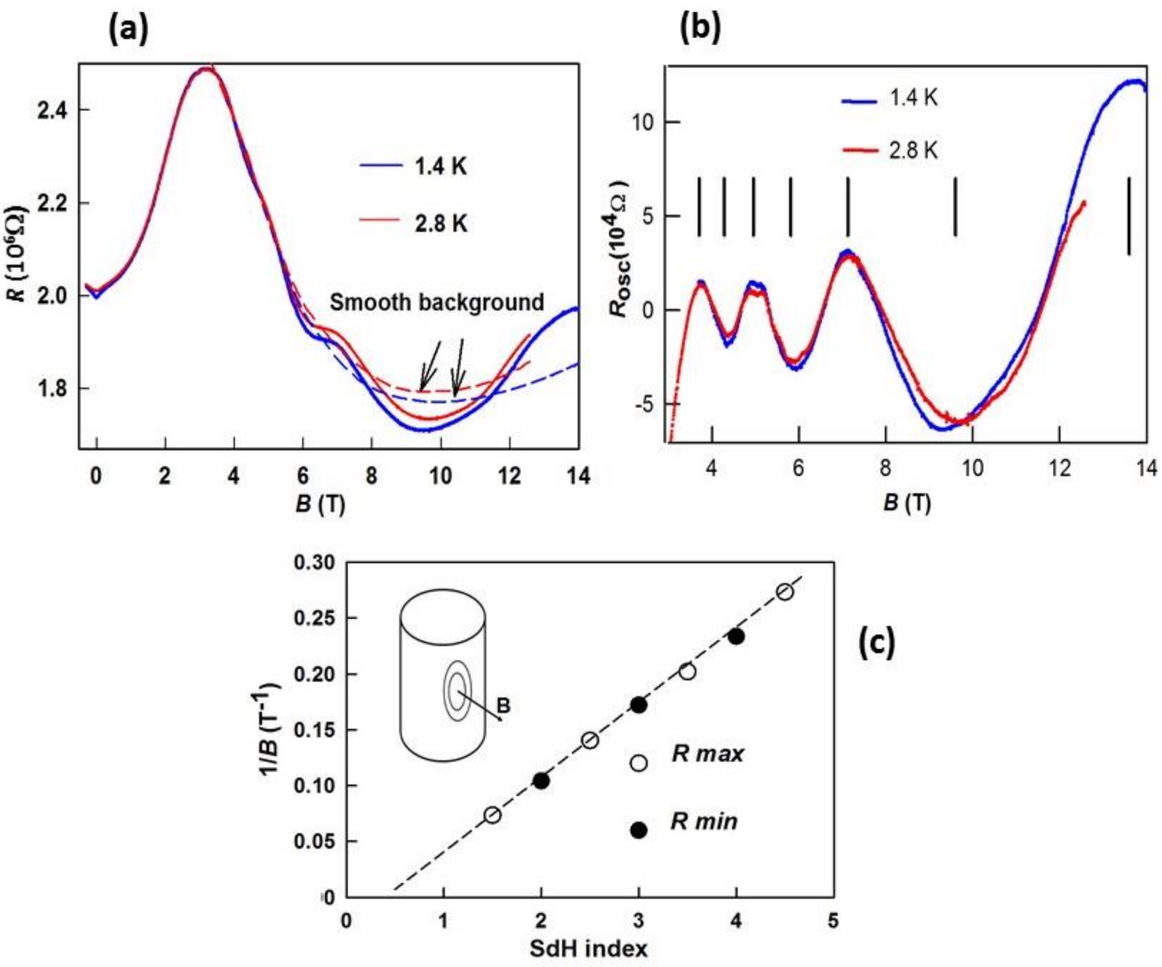

Supplementary Fig. 8 | Transverse magnetoresistance (TMR) of a 45-nm Bi nanowire showing Shubnikov-de Haas (SdH) oscillations. (a) The solid curves are the resistances of sample Q4 measured at 1.4 K and 2.8 K. The dashed curves, the smooth background, are the non-oscillatory parts extracted by filtering out the non-oscillatory parts using a fast Fourier transform algorithm. Temperature is indicated. **(b)** Oscillatory part of the resistance. (c) Landau-level fan diagrams for the SdH oscillations observed at 1.4 K; an integer n is assigned to minima of R (maxima of G). The dashed line indicates a linear fit. The inset illustrates the geometry of the nanowire and the assumed Larmor orbit, as well as the TMR magnetic field orientation.

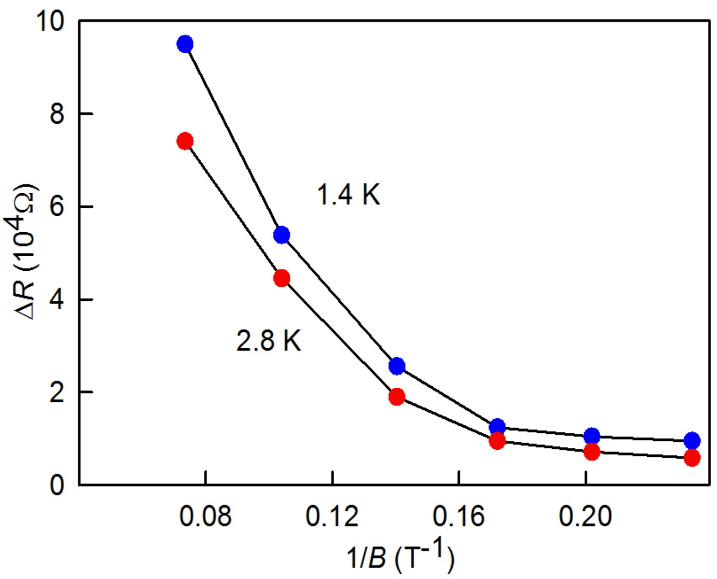

Supplementary Fig. 9. **Amplitude of the Shubnikov-de Haas (SdH) oscillation.** Temperature dependence of the amplitude Δ*R* of SdH oscillation.

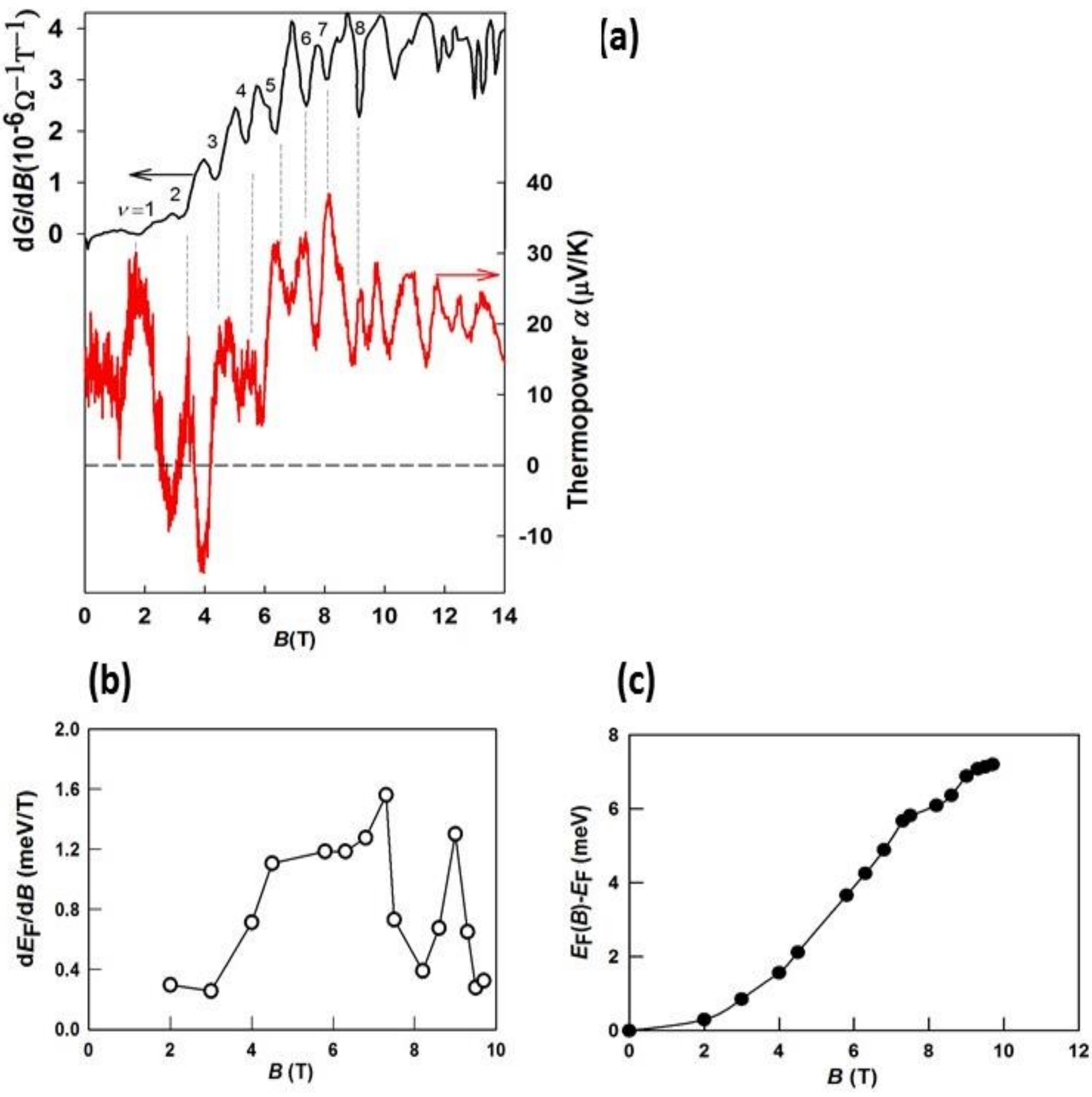

Supplementary Fig. 10. Aharonov–Bohm oscillations in a 50-nm Bi nanowire Q1 and Fermi energy versus magnetic field. a. Black and red represent dG/dB and thermopower α, respectively, as a function of B along the wirelength of sample Q1 at 1.5 K. The minima order ν is indicated. Inset, SEM cross-sectional image of the (50 ± 5)-nm wire (clear) in its glass envelope (grey background). **b.** Black and red represent dG/dB and thermopower α, respectively, as a function of B along the wirelength of the sample. The minima order ν is indicated. Inset, SEM cross-sectional image of the (50 ± 5)-nm wire (clear) in its glass envelope (grey background. **(b)** Derivative of the Fermi energy extracted from the experimental data in (a)

using: $\mathrm{d}E_{\mathrm{F}}\big/\mathrm{d}B = -\dfrac{\pi^2 k_{\mathrm{B}}{}^2 T}{3e}\dfrac{\mathrm{d}G\big/\mathrm{d}B}{G\alpha}$. Empty circles represent actual calculated values. **(c)** The Fermi energy change as a function of magnetic field.

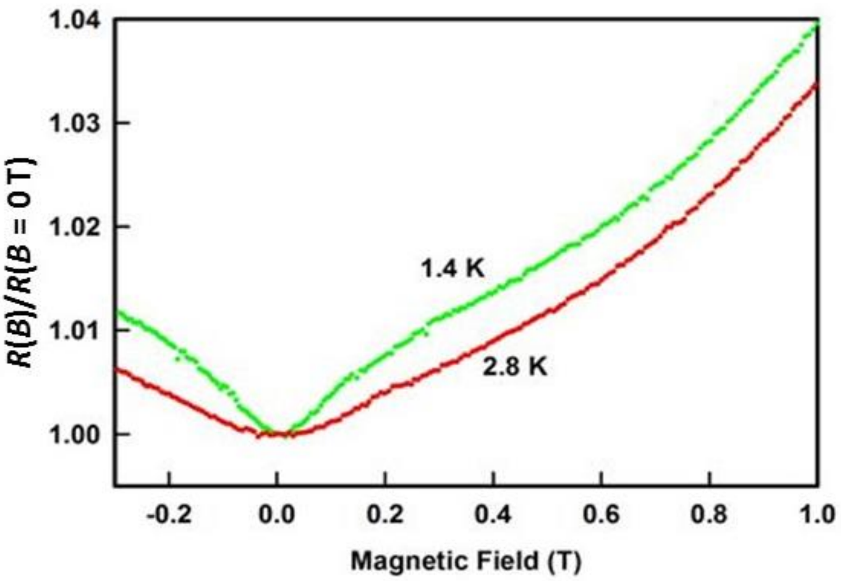

Supplementary Fig. 11. **Magnetoresistance as a function of transverse magnetic field B (field perpendicular to the wire axis), normalized to the resistance at zero field for 50 nm Bi nanowire Q4. The magnetic field is perpendicular to the wire axis.** The fluctuation of resistance that is observed correspond to rms(δG) <10^{-5} e^2/h and is interpreted as noise.

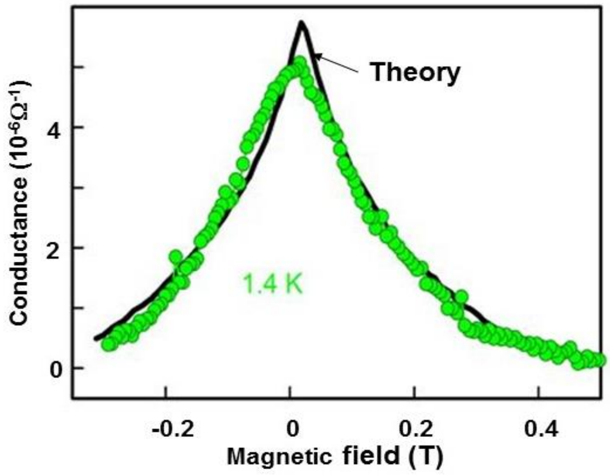

Supplementary Fig. 12. Transverse magneto-conductance (B perpendicular to the wire axis) around zero magnetic field at T=1.4 K shows a weak antilocalization effect. L_ϕ = 880 nm.
